# Infrared Thermally Enhanced 3-Dimensional Time of Flight Magnetic Resonance Angiography Imaging for the Visualization of the Arteries of the Face

**DOI:** 10.1093/asjof/ojaa020

**Published:** 2020-05-11

**Authors:** Benoit Hendrickx, Karl Waked, Marc Mespreuve

**Affiliations:** 1 Department of Plastic and Reconstructive Surgery, AZ Zeno, Knokke-Heist, Belgium; 2 Department of Plastic and Reconstructive Surgery, UZ Brussel, Jette, Belgium; 3 Department of Radiology, AZ Sint-Maarten, Mechelen, Belgium

## Abstract

**Background:**

The face is known for its extreme variation in vascular anatomy. Furthermore, the rapidly increasing number of filler treatments leads to an increase in severe filler-associated complications (such as skin necrosis and blindness) due to intra-arterial injection. Visualizing a patient’s individual complete facial arterial anatomy in a contrast- and radiation-free way has not been published before. This innovative imaging technique could, therefore, enhance the safety of minimally invasive surgical procedures as it provides a harmless way to map the arteries of the face.

**Objectives:**

Evaluate a newly developed imaging technique to visualize the arteries of the face in a noninvasive and radiation-free manner.

**Methods:**

The individual arterial facial anatomy of 20 volunteers was studied by an imaging technique, combining infrared (IR) facial warming and 3-dimensional (3D) time of flight (TOF) magnetic resonance angiography (MRA). The source and maximum intensity projection images were assessed by 2 investigators, familiar with the anatomy of the face.

**Results:**

The MRA technique visualized most of the main facial arteries, albeit in a variable way. The main facial branches of the external carotid artery (facial, angular, supralabial, and superficial temporal arteries) were illustrated well, whereas the visualization of the internal carotid branches (supratrochlear and supraorbital arteries) and nasal branches (dorsal nasal and lateral nasal arteries) was less consistent.

**Conclusions:**

The combination of IR “heat-induced enhancement” and a 3D-TOF MRA sequence may actually be an important step toward the visualization of the variable facial vascular anatomy in a noninvasive, radiation-free, and contrast-free manner.

The vascular anatomy of the face is extremely variable. Not only is the arterial course very tortuous but the localization of the facial arteries and their branches also varies significantly from person to person and even between both sides of the face.^1^ Performing filler injections in the face with standard text book anatomy in mind is therefore—although essential—in many ways obsolete as the arteries are rarely where they are expected.^2-4^ Due to the incremental use of soft tissue fillers (STF) for facial rejuvenation—mostly hyaluronic acid (HA)^5,6^—an increasing number of severe adverse reactions related to intravascular injection are observed.^7,8^ In spite of the potential reversibility of the inadvertent intra-arterial HA injection by injection of hyaluronidase,^9,10^ vascular occlusion may persist, especially in the area of the ophthalmic artery. Even in the hands of experienced plastic surgeons respecting the safety procedures,^11-13^ intra-arterial injection of STF may cause embolization leading to localized skin necrosis,^14^ blindness,^15,16^ or cerebral artery embolism.^17,18^ The most important danger zones for filler injections are localized in the central part of the face (lips, nose, nasolabial fold, tear trough, and forehead). The respective underlying arteries arise either from the external carotid system (facial [Fa], superior [SL] and inferior labial [IL], angular [Ang], lateral nasal [LN], infraorbital, and superficial temporal arteries) or the internal carotid system (supratrochlear [STr], supraorbital [SO], and dorsal nasal [DN] arteries, all arising from the ophthalmic artery). Each of these is prone to intravascular occlusion by fillers due to the uncertainty of the location of each artery in each individual. Occlusion of an artery feeding a skin territory will mainly lead to skin ischemia and subsequent necrosis, while intra-arterial injection in a vessel connecting to the ophthalmic artery and its branches may lead to blindness due to retrograde embolization and subsequent obstruction of the central retinal artery.^19,20^

In search of a harmless method for the visualization of the facial artery network,^21^ magnetic resonance angiography (MRA) seems to be the method of choice. A literature search and personal previous experiences—with and without gadolinium contrast—yielded, so far, no technique with adequate images of both the external and internal carotid arterial branches of the face with a clear visual distinction between arteries and veins.^22-24^ In order to procure a reliable image of the important superficial arteries of the face during facial surgical planning, we tested a combined technique of infrared (IR) facial heating and MRA (3-dimensional [3D]-time of flight [TOF] multiple overlapping thin slab acquisition [MOTSA]).

## METHODS

Between May and July 2019, the developed MRA-sequence was performed in 20 volunteers. A random selection of 20 volunteers was made out of the phone list of patients and coworkers. Our administration contacted these men and women randomly by phone and explained the purpose of our study. The first 20 people who were willing to participate in the study and who were compliant to the (limited) inclusion criteria were included in the study. No financial benefit was offered to the participants. The only inclusion criterion was aged between 18 and 65 years. Exclusion criteria were non-MRI-compatible internal devices (such as a pacemaker), metal plates in the face or skull, dental braces (but not dental wires), tattoos on the face (but not permanent make-up), claustrophobia, vascular disease, and congenital or acquired facial anomaly. Informed consent was obtained before the MRA examination. This study was approved by the ethical board of the hospital (EC 1904/B02620193974) and was fully conducted in the hospital of AZ Sint-Maarten in Mechelen (Belgium).

**Figure 1.**
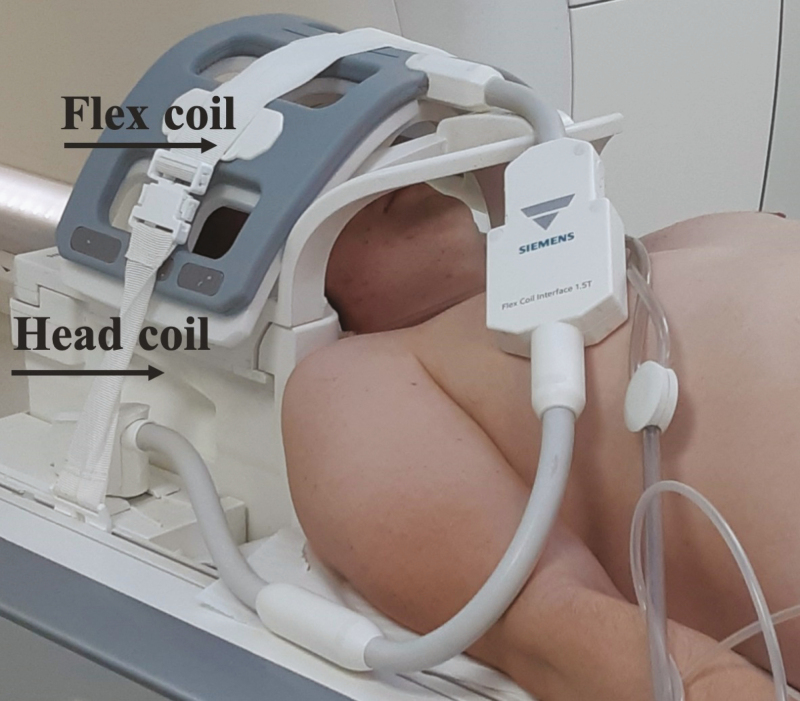
Position of the head and the flex coil for the MRA. A flexible wrap-around 4-channel surface coil may be mounted on top of the head coil in order to increase the signal reception from the facial arteries. Illustration of the setup on a 58-year-old male patient. MRA, magnetic resonance angiography.

As still more than 60% of all MR scanners globally are 1.5 Tesla machines, all images were acquired on a 1.5 Tesla full-body MR system (Magnetom Aera Siemens, Erlangen, Germany), using a dedicated 20-channel head coil. Additionally, a flexible wrap-around 4-channel surface coil was mounted on top of the head coil (Figure 1) in order to increase the signal reception from the facial arteries. Before the TOF MRA examinations, which are known to be flow dependent, all volunteers were positioned with closed eyes in the front of an IR light source (Philips PR 3120 of 300 W with an Infracare screen, which filters out the UV light) at a distance of 30 cm and with their face parallel to the lamp during 10 minutes, as we supposed that this would induce vasodilatation and enhance the vascular flow. At the same time, they were asked to stimulate their facial muscles by slowly moving their lips and forehead and switching between several facial expressions during the exposure time in order to further increase the blood flow, thanks to muscle activation. In order to truly appreciate the benefit of IR exposure, a second 3D-TOF MOTSA scan was performed in a random selection of 5 patients without IR exposure.

**Figure 2.**
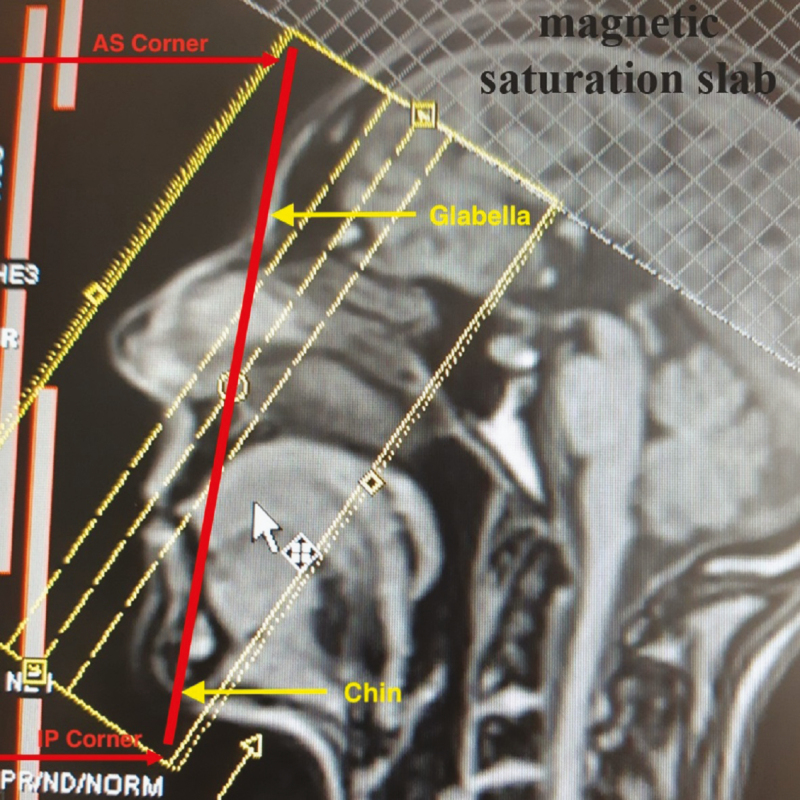
The positioning of the 3D-TOF MOTSA slabs block on the localizer. The red line drawn from the glabella to the chin transects the slabs position block (yellow rectangle) from the anterosuperior (AS) corner to the inferoposterior (IP) corner. A magnetic saturation slab is positioned above the slabs block. TOF, time of flight; MOTSA, multiple overlapping thin slab acquisition.

**Figure 3.**
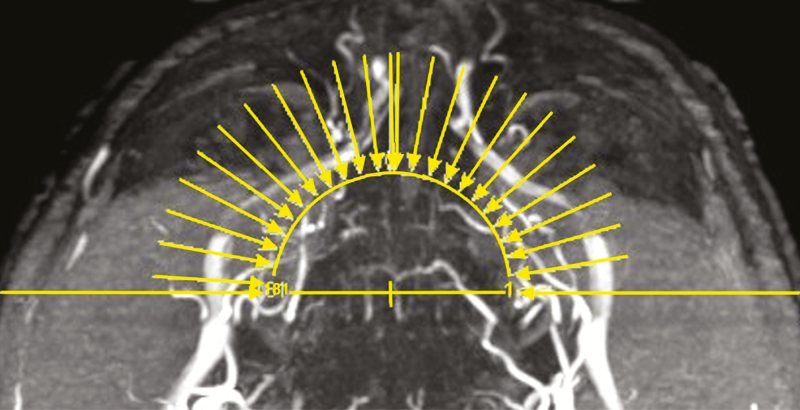
The MIP—reconstruction levels shown on the axial view for the same 58-year-old male patient featured in Figure 1 and 4. MIP, maximum intensity projection.

After the acquisition of the scout views, a 3D-TOF MOTSA MRA sequence was acquired in an oblique coronal plane (tilting of 25° backward vs the line between the glabella and the chin) (Figure 2). The MRA protocol is summarized in Table 1. The acquisition time was 16 minutes and 14 seconds. During that time, the patient was asked to remain completely still. A multislab technique was used to reduce the saturation effect of the inflowing blood signal. Maximum intensity projection (MIP) images were made every degree over 180° in a sagittal plane (Figure 3). 

**Table 1.** 3D TOF MOTSA Sequence

TR	30	msec
TE	6.8	msec
Acquisitions	1	
FOV	180	mm
Flip angle	30	°
Matrix	180 × 180	pixels
Slice thickness	0.5	mm
Averages	2	
SNR	1.0	
Voxel size	0.4 × 0.4 × 0.5	mm
Time of acquisition	16 min 14 sec	

Gradient echo sequence with 5 overlapping (17.5%) slabs; FOV, field of view; MOTSA, multiple overlapping thin slab acquisition; SNR, signal-to-noise ratio; TE, time of echo; TOF: time of flight; TR, time of repetition.

The source and MIP images were randomized and then independently assessed by an experienced radiologist and a plastic surgeon, familiar with the facial anatomy (M.M. with 32 years and B.H. with 15 years of experience). The images were displayed on a medical monitor, 6 Megapixel Barco display, 32″, 3280 × 2048 resolution (Barco / Kortrijk, Belgium).

A total of 18 arteries (9 on the right and 9 on the left side of the face) were assessed. Vessels obscured by metal artifacts (around the mouth, due to dental wires, or in the cheek, due to metal screws) were eliminated from the analysis in order to obtain a strictly technical evaluation of the IR-TOF sequence.

The visualization of the important superficial facial arteries (facial [Fa], Ang, SL and IL, LN, DN, STr, SO, and superficial temporal [ST] arteries) was scored on a 4-point scale: 0, artery not visualized; 1, less than 50% of the artery visualized; 2, more than 50% of the artery visualized; 3, artery entirely visualized.^3^ In case that the same artery was scored in a different way by both assessors, the MRA was analyzed by both investigators together and a decision was obtained by consensus. Interrater reliability was evaluated using Cohen’s kappa value (<0: no agreement; 0-0.20: slight agreement; 0.21-0.40: fair agreement; 0.41-0.60: moderate agreement; 0.61-0.80: substantial agreement; 0.81-1.00: full agreement). We assumed that the voxel size of the implemented sequence (0.4 × 0.4 × 0.5 mm) was small enough to visualize each superficial facial artery, as the mean diameter is <2.5 mm, according to anatomical studies.^25^ An artery is fully visualized (score of 3) when the full (anatomical) course is visible on the native MRI images. For the Fa, this means a visible course until the inferior alar border, for the Ang until the medial canthus of the eye, for the SL and IL until the midline of the face, for the DN and LN until a visible anastomosis between both arteries, for the Str and SO until mid-height of the frontal scalp (Str, SO), and for the ST until the medial border of the temporal fossa. 

It was a conscious decision of the investigators to not include a comparison with classic angiography (which is still considered as the golden standard for arterial visualization) or CT angiography (CTA), as both imaging techniques involve an invasive procedure, might induce an allergic reaction by injecting a contrast medium, and may potentially be harmful to the patient due to exposure to radiation.

Hence, the aim of this study was purely to evaluate a completely harmless technique for facial artery visualization. A comparison with an invasive imaging method is beyond the scope of this study and would not be acceptable on ethical grounds for the purpose of filler injections.

## RESULTS

Of the 20 volunteers, 14 were men and 6 were women, with a mean age of 42.9 +/− 12.3 years (range, 28-65 years). The mean BMI was 24.3 (range, 19.2-31.4). Eight patients had dental wires (after having braces), which surprisingly did not necessarily lead to perioral artifacts. In 2 patients, the perioral vessels were absent (no interference of metal artifacts in participant 5 on the right side with an absent SL and IL arteries and in participant 15 there as an absent SL artery on the left side). One patient had a big artifact of the left cheek, of which we later found out that he has a metal screw in his mandibula after being involved in an accident.

**Figure 4.**
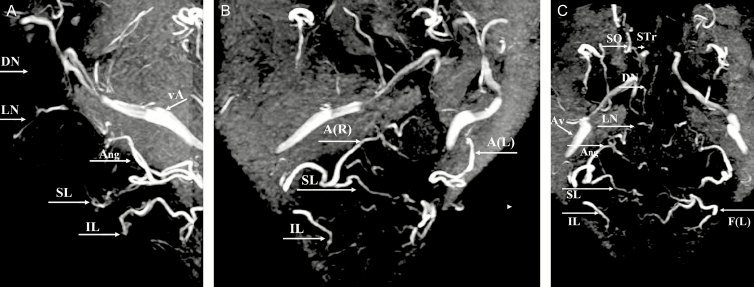
MRA findings (MIP of 3D-TOF) in a 58-year-old male patient. (A) Lateral view. (B) Right oblique view with details of the labial arteries and right (R) and left (L) angular arteries A(R) and A(L). (C) Anteroposterior view of the (annotated) arteries. Superior (SL) and inferior labial artery (IL), angular artery (Ang), lateral nasal artery (LN), dorsal nasal artery (DN), supratrochlear artery (STr), supraorbital artery (SO), facial artery (F), and angular vein (vA). MIP, maximum intensity projection; TOF, time of flight.

Of the 20 participants, 19 completed the whole MRA protocol successfully. Our first volunteer had to be eliminated due to a technical problem (wrap-around surface coil got disconnected during the examination and the volunteer was not willing to undergo a second examination). In spite of the rather long examination time, we did not notice significant motion artifacts. Although the vasodilation and skin redness due to the IR exposure were visible, no adverse reactions due to the “IR enhancement” were observed nor mentioned by the patients. The warmth sensation remained for an average of almost 2 hours. The overall visualization of the arteries was remarkably good in some participants (Figure 4). The only (sometimes confusing) venous structure—running more posteriorly and laterally outside of the field of interest—was the large Angular Vein (Figure 4C). Arteries (partially) masked by dental wires were evaluated but eliminated for the calculation of the global arterial result: 7 Ang (4 of the right hemiface and 3 of the left hemiface), 15 SL (7 of the right hemiface and 8 of the left hemiface), and 16 IL (7 of the right hemiface and 9 of the left hemiface) arteries. In a detailed analysis per artery (Tables 2 and 3), the grade of visibility was less for the SO (mean, 1.0; median, 1), DN (mean, 1.3; median, 1), and STr (mean, 1.5; median, 1) arteries. The IL (mean, 1.8; median, 1) and ST (mean, 1.9; median, 2) arteries were in the middle group. The Ang (mean, 2.0; median, 2), Fa (mean, 2.3; median, 3), LN (mean, 2.1; median, 2), and SL (mean, 2.3; median, 2) arteries were most constantly illustrated. With a general Cohen’s kappa value of 0.80, the interrater reliability was at the upper limit of the substantial range. No specific general parameter (age, gender, weight, and smoker) influencing the quality of the arterial visualization could be clearly identified, maybe due to the limited number of participants.

**Table 2.** Individual Visual Scores for the Main Arteries (Right and Left) of the Face

	Right									Left								
P	SO	STr	DN	LN	Ang	ST	SL	IL	Fa	SO	STr	DN	LN	Ang	ST	SL	IL	Fa
1	1	1	1	1	0	1	1	1	1	0	1	1	1	2	1	3	1	2
2	0	2	0	1	2	2	2	1	1	0	2	0	1	1	2	2	3	2
3	0	2	3	2	2	2	3	2	3	0	1	2	1	0**	2	0**	0**	0**
4	0	0	1	2	1	2	2	2	2	0	0	0	2	1	2	2*	1*	2
5	0	1	2	2	1	1	0*	0*	0	1	1	3	3	1	1	2	1	3
6	0	1	0	1	0*	3	0*	0*	0*	3	3	0	0	0*	2	0*	0*	0*
7	1	1	1	3	3	3	2*	1*	3	1	1	1	3	3	3	1*	1*	3
8	0	0	1	3	3	2	3*	1	3	0	0	3	3	3	1	3	1	3
9	1	1	1	1	3	3	1*	1*	3	1	2	0	1	1	3	0*	0*	3
10	1	1	1	1	3	1	3	3	3	1	1	1	1	2	0	1	3	1
11	2	2	1	1	0*	3	1*	1*	3	1	1	1	1	0	3	0*	0*	3
12	1	1	1	2	0*	0	1*	1*	1	1	1	2	2	1*	1	1*	1*	2
13	0	0	1	3	2	3	3	1	3	1	1	1	1	1	3	1*	1*	2
14	3	3	2	3	1*	3	1*	1*	1	3	3	2	3	2	3	3	0*	3
15	2	3	1	2	1	2	2	1	1	2	3	2	2	2	2	1	1	1
16	1	1	1	3	2	1	1	1	2	1	1	0	3	2	1	3	1	3
17	2	3	2	3	3	1	1	1	2	0	1	3	3	3	1	3	1	3
18	2	2	2	3	3	2	2	1	3	1	2	2	3	3	3	2	1	3
19	2	3	3	3	3	1	3	3	3	2	2	2	3	3	1	3	3	3

Arteries (partially) masked by dental wires (*) and by a metal screw (**) were evaluated, but eliminated for the global arterial score: 7A, 15 SL, and 14 IL. Note that the first participant had to be eliminated because of a technical issue. ANG, angular artery; DN, dorsal nasal artery; FA, facial artery; IL, infralabial artery; LN, lateral nasal artery; P, participant; SL, supralabial artery; SO, supraorbital artery; ST, superficial temporal artery; STR, supratrochlear artery; 0, not visualized; 1, less than 50%; 2, more than 50%; 3, completely visualized.

**Table 3.** Global Visual Score for the Main Arteries of the Face

Artery	SO	STr	DN	LN	ST	Ang	SL	IL	Fa
Mean	1.0	1.5	1.3	2.1	1.9	2.0	2.3	1.8	2.3
SD	0.9	1.0	0.9	0.9	0.9	1.0	0.8	0.9	0.9
Median	1	1	1	2	2	2	2	1	3
Range	0-3	0-3	0-3	0-3	0-3	0-3	1-3	1-3	0-3
*n* =	38	38	38	38	38	31	23	22	35

Arteries (partially) masked by dental wires were eliminated: 7A, 15 SL, and 16 IL. ANG, angular artery; DN, dorsal nasal artery; FA, facial artery; IL, infralabial artery; LN, lateral nasal artery; SD, standard deviation; SL, supralabial artery; SO, supraorbital artery; ST, superficial temporal artery; STR, supratrochlear artery.

**Figure 5.**
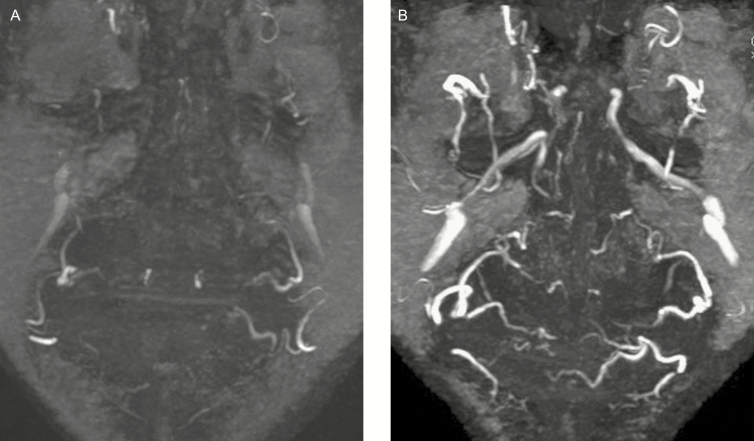
MRA findings (MIP of 3D-TOF) of a non-heated vs heated face. (A) Non-heated face without previous infrared exposure. (B) Heated face with previous infrared exposure. The Infrared-exposure results in a far better visualization of all facial vessels, with a larger caliber of the arteries and a higher visual signal. MRA, magnetic resonance angiography; MIP, maximum intensity projection; TOF, time of flight.

In a random selection of 5 patients, a second MRA was taken without previous IR exposure. Figure 5 shows a comparison of a 3D-TOF MOTSA MRA without (A) and with (B) previous IR exposure. The effect of IR exposure—vasodilation and increased flow—clearly results in a higher number of visible arteries with a larger diameter and higher signal strength.

## DISCUSSION

### 3D-TOF Compared With Other Imaging Techniques

There is a high need for information on the individual arterial anatomy in facial rejuvenation procedures with STF due to the increasing number of vascular complications.^19,26^

Taking into account the fact that a filler injection remains an aesthetic procedure, developing a risk-free, radiation-free, contrast-free, and noninvasive imaging technique to visualize anyone’s individual full facial arterial anatomy is mandatory. This is—to the best of our knowledge—the first description of such a technique.

The most precise evaluation technique for the facial arteries remains conventional angiography (CA) using contrast medium. However, the risk of a new focal neurological deficit caused by CA ranges between 0.14% and 0.5%.^27^ This means that up to 1 in 200 patients may suffer from a cerebral attack during a CA. With more than 10 million filler injections performed annually worldwide, it would not be acceptable to perform CA in such a large patient population, despite its higher resolution compared with MRA. CTA is also a very useful tool to illustrate the 3D structure of the vessels, but there is an additional risk of complications due to the use of iodine contrast medium and the radiation exposure (the overall patient risk per CTA procedure ranges between 15 and 36 cancer risks per 1 million procedures).^28^ In the light of these serious medical complications, we decided not to compare our results to CA or CTA, as approval would not be granted by the ethical committee.

Apart from MRA, ultrasonography (US) is the only other noninvasive imaging technique. However, the US cannot visualize a wide area nor the 3D structure of the vasculature; it is very time-consuming and operator-dependent.^29^ Moreover, obtaining a complete 3D overview of the facial vascular anatomy using the US is currently not possible and the examination has to be repeated before each filler injection as no stereo-tactile information is obtained, implying that the images have to be generated “on the spot” every time. 

Besides, an MRI provides much more than just vascular information. Muscles, salivary glands, skin surface, and more can be visualized. This may all be valuable information with regard to minimally invasive procedures.

MRA may use either vessel flow or IV contrast to enhance the vessels. The latter method needs an IV gadolinium injection, which again entails potential complications for the patient and is preferably avoided with regard to aesthetic procedures. On the contrary, in bright-blood methods, the signal from the moving protons is accentuated relative to the stationary protons of the surrounding tissue.^23,30^ These flow-based techniques include mainly TOF, Phase Contrast (PC), and Fast Spin Echo Imaging (FSE).^22,31^ TOF is the most time-efficient method for obtaining MRA images.^32,33^ A single measurement is performed, with the stationary tissue signal suppressed relative to the flowing tissue signal.^34^ MIP images are used to visualize the MRA data.

TOF MRA has never been successfully used for the visualization of all the facial blood vessels,^35^ mainly due to the tortuous arterial courses along different planes and directions, which challenges the use of TOF to a greater extent. As the branches of the facial arteries have complicated courses and relatively slow flow rates, we tried to accelerate the flow and enhance signal strength by heating the superficial arteries of this region, as well as activating the muscles around them. Warmth application including IR radiation has been used for pain therapy for many years. The skin penetration depends on the wavelength. Only high IR-A content waves can reach the vascular layers of the subcutis and can increase the local circulation significantly. A PR 3120 Infracare lamp for home application (Philips, Eindhoven, The Netherlands) produces around 50% of IR-A. A single exposure of 10 minutes at 30 cm distance remains far below the daily therapeutic duration of 15 to 30 minutes and can, therefore, be considered as harmless. However, people with swelling or inflammation should not be exposed to this device as heat may aggravate their complaints. The recommended distance between the lamp and the skin should be respected. Also, the eyes should be closed as a prolonged exposure to the IR lamp may induce corneal damage.^36^ The effect may further be optimized by holding the face parallel to the lamp in order to warm every facial region evenly.

In this study, the advantage of using IR exposure was so obvious, that we applied it on all other test patients. The combination of warmth of the IR lamp and muscle activation during IR exposure resulted in vasodilation and an increased blood flow, both important factors in obtaining a better 3D-TOF MRA image quality. Vasodilation results in larger vessel diameters but might decrease the blood flow velocity. However, the simultaneously occurring increase in the flow speed also results in a better 3D-TOF signal strength. The comparison in 5 patients with and without IR exposure confirmed this assumption. Important to notice, as well, is the fact that the location of the arteries did not change due to the facial heating, only the flow speed and diameter of the vessels increased.

This finding may give rise to the suggestion to cool down the face before filler injections, in order to induce vasoconstriction of the facial arteries and, therefore, decrease the risk of intravascular injection. The native MRI (Figure 5A, which shows the MRA of a face before facial warming) may be considered as an example of a cooled down face, where the size and number of visible arteries are clearly diminished, compared with an MRA after facial warming (Figure 5B). An effective cooling down method may be exposure to cold air from an air conditioner or by means of a cold pack. Cooling down the skin may have an additional benefit of decreasing the pain sensation during filler injections. As arterial structures are already poorly visible in non-preheated patients, these suggestions may be hard to objectivate, however.

### MRA Findings

We noted that the SL and, especially, the IL arteries were (partially) obscured by metal artifacts due to remaining dental wires (15 SL—7 of the right hemiface and 8 of the left hemiface) and 16 IL (7 of the right hemiface and 9 of the left hemiface) were eventually eliminated from the analysis, in order to truly focus on the technical evaluation of the IR-TOF sequence.

The grade of visibility was lower for the short path of the small and/or tortuous DN, SO, and STr arteries.^37^ This might be related to the fact that for this imaging sequence, the angle between the slice plane and the direction of the vessels has a great effect on the inflow phenomenon and is related to the degree of the visualization of the vessels. As the DN, SO, and STr arteries run almost parallel to the slice plane, their signal strength on TOF imaging might be decreased. Furthermore, the mobility of the skin in this region is more limited, which may be an influencing factor during the “muscle activation” phase during IR time. Equally striking was the fact that, in the participants in which the visualization of these most cranially located vessels was better, the visualization of the more caudally located SL and IL arteries was remarkably degraded. This may suggest that the workflow could benefit from a more rigorous control of the strict parallel position of the face relative to the IR lamp during the exposure. The advantage of scanning in an oblique coronal plane has also partially resolved these issues.

The middle group (IL and especially ST) arteries are positioned more posteriorly and, in the case of the ST arteries, also more lateral on the face. Hence, they are less influenced by the IR warmth effect. A longer exposure time and/or even a flow enhancement by 2 IR devices bilaterally placed in an oblique way could be considered in order to increase the visualization of these vessels.

The Ang, Fa, and SL arteries were best illustrated, which can be explained by their central and frontal location, as well as their vessel size. The usually very small caliber LN artery was also visualized in the majority of patients, which may be explained by the fact that its blood flow directions run perpendicular to the scanning direction.

Providing a 3D-visualization of the facial arteries in a noninvasive, radiation-free, and contrast-free way may lower the threshold to perform this kind of imaging on a more frequent basis. Knowledge of the exact location of a patient’s facial blood vessels may be beneficial in all kinds of surgery: from free flap surgery to locate potential acceptor site vessels (such as the ST arteries), to reconstructive surgery with local flaps that can be tailored in such a way to include a specific vessel, and even minimally invasive surgery to localize and avoid important arteries during rejuvenation techniques such as filler injections. Even though other imaging techniques, such as CA and CTA, might be the better choice for reconstructive procedures, in case of a contra-indication (such as contrast medium allergy), this 3D-TOF imaging sequence may be an alternative. However, as severe complications generally arise secondary to intravascular injection (embolia medicamentosa) of filler injections, the IR-MRA visualization of these arteries may help to prevent these serious complications by altering the puncture site or the injection path to avoid the arteries. Hence, the MIP reconstructions provide an added benefit of showing a 3D reconstruction of the facial contour, therefore helping the injector to estimate the location of a certain artery. Additionally, the radiologist may place some landmarks or measurements at certain (frequent) filler injections sites to annotate the vicinity of an artery.

Furthermore, in aesthetic procedures, we would truly advocate the use of a risk-free imaging technique, as any form of the invasive procedure (contrast injection) or radiation exposure (CTA) should be avoided.

### Limitations

The biggest limitation of our study is that our IR MRA 3D-TOF technique was not compared with CA or CTA using a contrast medium. Both latter imaging techniques would indeed offer a higher resolution of the angiographic assessment, but they both imply significant potential complications to the test population and were, therefore, not suited as a control in this study. The aim of our study was also not to compare this innovative technique to the gold standard, as they each have a different purpose and CA, nor CTA should never be used for the mapping of the arteries of the face for cosmetic purposes. For the preoperative planning of reconstructive procedures, one could still consider using CA or CTA, as the better resolution justifies the potential risks of these imaging methods. However, in planning facial filler injections, we believe that a non-risk imaging technique is primordial, despite its lower resolution.

To have a more thorough evaluation of the MRA findings, an imaging validation with ultrasound could be implemented, where each volunteer would undergo a complete ultrasound mapping of the arteries of the face in order to truly confirm the presence or absence of certain arteries that are not visible on the MRA. However, performing such an ultrasound would take at least a double or triple amount of time, compared with an MRA. The heat effect, which dilates the arteries, would, therefore, also be lost during the examination (due to both the length of the procedure and the fact that the gel is a cold substance, causing vasoconstriction), which would make the visualization of the arteries in a number of areas (such as the SO and Str arteries) even more difficult. Additionally, the purpose of this study was not to compare this new MRA sequence to already established imaging methods, but merely to describe this new protocol for a noninvasive visualization of the entire arterial network of the face and thus to evaluate the usefulness and applicability of an MRA for cosmetic purposes.

A known limitation of MRA is the artifacts due to metal implants, such as screws or plates. As expected, dental wires did slightly interfere with the overall image quality, but this was not a constant finding. As mentioned before, claustrophobia was an exclusion criterion for participation in this study, although we do believe that this group of people only represents a small minority of the filler population.

As the arterial anatomy is not known to change over time in the absence of major trauma or surgery, a single MRA can provide guidance to the injector for many years. The arteries might become somehow more tortuous but will not change in location.^38^ Facial surgery or trauma might, however, necessitate a new MRA to verify the location of the arteries.

Parallel imaging was not tested in this study, as we aimed to develop a technique that could be used on widespread common 1.5 T MRI systems. If available, however, parallel imaging may indeed reduce the examination time and reduce motion artifacts. Further tests and extension to 3T MRI systems may also improve this technique.

We realize that, in some countries, the indication to perform an MRA for aesthetic purposes may not fall under reimbursement policy; patients or practitioners may, therefore, need to take into account the cost of this MRA. However, we truly believe that the added benefit of knowing the location of the individual arterial anatomy of each patient largely outweighs the cost and effort of a 1-time MRA, of which the findings may be useful for multiple years of repeated filler treatments. This effort might even be more justified if the patients would be correctly informed on the risk of facial filler injections, before a treatment.

## CONCLUSIONS

Our initial experience shows that the combination of IR “heat enhancement” and an MRA 3D-TOF sequence makes it feasible to visualize a number of facial arteries in a radiation-free, contrast-free, and noninvasive way. With this risk-free technique, we fill in the high need for information about the individual patient’s anatomy to better plan and execute any sort of facial procedure, especially filler injections.
